# Self-assembling amphiphilic peptides†

**DOI:** 10.1002/psc.2633

**Published:** 2014-04-13

**Authors:** Ashkan Dehsorkhi, Valeria Castelletto, Ian W Hamley

**Affiliations:** Department of Chemistry, University of ReadingWhiteknights, Reading, RG6 6AD, UK

**Keywords:** self-assembly, amphiphilic peptides, surfactant-like peptides, amyloid peptides

## Abstract

The self-assembly of several classes of amphiphilic peptides is reviewed, and selected applications are discussed. We discuss recent work on the self-assembly of lipopeptides, surfactant-like peptides and amyloid peptides derived from the amyloid-*β* peptide. The influence of environmental variables such as pH and temperature on aggregate nanostructure is discussed. Enzyme-induced remodelling due to peptide cleavage and nanostructure control through photocleavage or photo-cross-linking are also considered. Lastly, selected applications of amphiphilic peptides in biomedicine and materials science are outlined. © 2014 The Authors. *Journal of Peptide Science* published by European Peptide Society and John Wiley & Sons, Ltd.

## Introduction

Peptides are short oligomers made from amino acids linked via amide bonds. Amino acids are the natural building blocks that make up both proteins and peptides. Amino acids consist of an amine, carboxylic acid and functional groups as well as distinctive side chains. There are approximately 20 different amino acids, with 20 different side chains, which give rise to their unique characteristics and variability. Differences in the amino acid side chains allow them to be grouped into different categories according to their chemical composition such as hydrophobic/hydrophilic/aliphatic/aromatic or neutral/positively/negatively charged. The numerous interactions between amino acids allow for complex self-assembled nanostructures to develop. These types of interactions include hydrogen bonds, electrostatic interactions, hydrophobic interactions, aromatic interactions (*π–π* stacking) and van der Waals forces. The varying properties of amino acids can therefore be manipulated in the design of peptides for initiation of self-assembly to produce novel functional biomaterials.

Self-assembly of biomolecules is the ability to associate via noncovalent interactions into ordered 3D structures through a bottom-up approach without the guidance of an external source. Self-assembly occurs naturally in living cells; examples include the self-assembly of lipids in the formation of cell membranes, protein folding in enzymes, formation of the DNA double helix stabilised by intermolecular hydrogen bonding, formation of viruses (protein capsids wrapped around a nucleic acid core), microtubules involved in cell division and flagella in bacteria, which aid in movement. Self-assembly is an important process in nature and has inspired many in the field to exploit such remarkable processes in the creation of enhanced biomedical materials. Although the noncovalent interactions involved in self-assembly have a much lower energy than covalent bonds, it is sufficient to produce highly organised and robust nanostructures.

Peptide amphiphiles (PAs) have gained a huge amount of attention over the past decade because of their ability to self-assemble into a range of novel nanostructures. Their self-assembling abilities are dictated by their amphiphilic nature owing to the inclusion of a lipid chain attached to a biofunctional peptide epitope that can participate in secondary structures such as *β*-sheets. The unique interplay of intermolecular hydrogen bonding along with hydrophobic and electrostatic interactions leads to well-defined self-assembled nanostructures 1–7.

Different classes of amphiphilic peptides include the following: (i) peptides that consist of both polar and nonpolar residues, giving rise to both hydrophobic and hydrophilic properties 6, (ii) hydrophilic peptides attached to hydrophobic lipid alkyl chains 2,6,8 and (iii) peptide-based copolymers 2. The work presented in this review will primarily focus on the self-assembly of PAs (lipidated peptides) along with amphiphilic peptides (oligopeptides comprising both hydrophobic and hydrophilic features).

Peptide amphiphiles are peptide-based molecules that have the tendency to self-assemble into high-aspect-ratio nanostructures under certain conditions of pH, temperature and ionic strength. Lipopeptide PAs consist of two main regions. The first is a hydrophobic alkyl tail or lipid chain, which is attached to a hydrophilic peptide sequence forming the PA head group 3–5. In aqueous solutions, the aggregation of hydrophobic tails drives self-assembly, resulting in the presentation of bioactive peptides on the surface of the nanostructure 1–3,6. Self-assembly of PAs occurs as it is necessary for the hydrophobic alkyl chain to be screened from the aqueous environment 5,7. The peptide epitope usually comprises a biologically derived motif, which can play an important role in biological processes such as cell signal transduction, cell adhesion in the extra-cellular matrix (ECM), cell growth and cell mobility 5,6,9. Apart from fibrils, PAs can spontaneously self-assemble into a range of other self-assembled structures such as spherical micelles, vesicles, bilayers (lamellar structures), nanofibres, nanotubes and ribbons 2,10–12.

Reports on PA nanofibres as biomimetics that can be applied *in vivo* include studies by Stupp and co-workers. These include PAs that can be used in regenerative medicine for cartilage regeneration 4, to treat ischaemic tissue disease 5 and neuronal fibre damage 13 and to assist in neuronal differentiation 14 and angiogenesis 15. PAs can be utilised to act as therapeutic agents to treat diseases by transporting hydrophobic drugs to a specific site as they can be metabolised and biodegraded into lipids and amino acids, which can easily be removed in the kidneys 4. This is performed by the incorporation of the hydrophobic tail, which can travel across cell membranes and increase bioavailability, while the peptide epitope can be used to target a specific cell by a ligand–receptor complex 1. Other important applications of PAs have been proposed such as use in antimicrobials 16–18, cell culture scaffolds for tissue engineering, skincare and cosmetics 19–21, gene delivery, templates for biomineralisation 22 and stabilisation of membrane proteins.

**Biography**

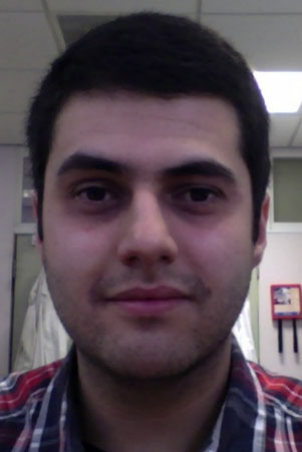
Dr Dehsorkhi received his undergraduate degree in Biomedical Sciences from Queen Mary, University of London, in 2010. He completed his PhD in Chemistry at the University of Reading under the guidance of Professor Ian W. Hamley. His work is focused on the self-assembly mechanism of peptide amphiphiles using a range of biophysical techniques. He is currently a postdoctoral research associate working on a Biotechnology and Biological Sciences Research Council-funded project with both Professor Ian W. Hamley and Dr Che J. Connon at the University of Reading, to produce a bioprosthetic cornea, based on an orientable hydrogel template using self-assembling peptide systems.

**Biography**

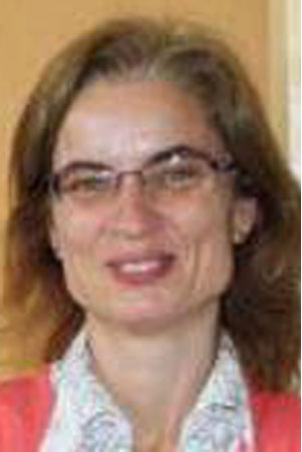
Valeria Castelletto received her PhD in Condensed Matter Physics from the University of São Paulo (Brazil). Following her PhD, she continued to study the structure of soft materials during her work at the National Synchrotron Laboratory (Brazil), the Laboratoire Léon Brilloun (France) and the École Normale Supérieure de Paris (France). Valeria moved to the UK to work at the University of Leeds to study the structure and dynamics of block copolymer and peptide systems. She conducted further research on peptide biomaterials at the University of Reading (UK). She joined the Biotechnology Group at the National Physical Laboratory (Teddington, UK) in 2014. Her current research interests are focused on soft materials, in particular in the areas of peptide biomaterials, enzymes and proteins.

**Biography**

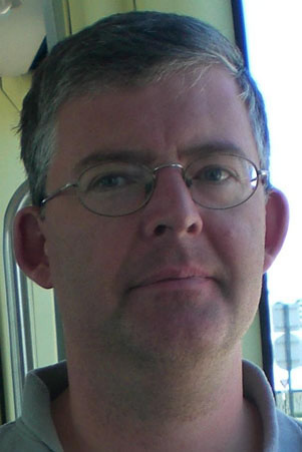
Professor Ian W. Hamley is Diamond Professor of Physical Chemistry at the University of Reading. He has more than 20 years' experience of research on different types of soft materials, including peptides, polymers, liquid crystals and surfactants. He obtained his PhD from the University of Southampton in 1991 and then undertook postdoctoral research at AMOLF, Amsterdam, and the University of Minnesota. In 1993, he returned to a lectureship at the University of Durham, UK, and moved to the University of Leeds in 1995 where he was promoted to a professorship in 2004. He relocated to the University of Reading in 2005. He received a Royal Society-Wolfson Research Merit Award, commencing in 2011. His research programme on amyloid-forming peptides and peptide copolymers focuses on their self-assembly behaviour. He has published over 300 journal publications (*h*-index 51) and several edited and authored books.

## Design of Self-assembling Peptide Amphiphiles

**Figure 1:**
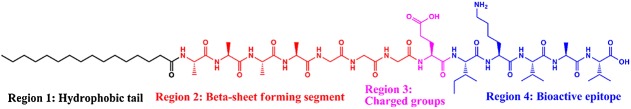
The four domains in a PA molecule that are required for self-assembly in a functional system 8.

In order for a fully functional PA to assemble into a well-defined supramolecular structure, it requires four essential domains as shown in Figure 1. These domains are (i) a hydrophobic tail to form hydrophobic interactions, (ii) a peptide sequence that is able to form intermolecular hydrogen bonds, which determines the interfacial curvature of the self-assembled structure, (iii) charged amino acids to promote solubility and (iv) a functional peptide epitope 8.

The most important aspect of a PA molecule is its amphiphilicity, which is the driving force for self-assembly. The hydrophobic tail is responsible for driving self-assembly and exposing the functional peptide group on the surface of the nanostructure 6,8,9. The peptide sequence must have the ability to form intermolecular hydrogen bonds, leading to *β*-sheet formation, allowing for further aggregation to occur 3,6,23. The interplay between hydrogen bonding and hydrophobic interactions allowing self-assembly of PAs has been probed in studies on PAs with extended alkyl chains 24. The charge of an amino acid within the peptide sequence can influence the solubility of PAs. Therefore, changing the solution pH can substantially alter self-assembly 8,10,25.

## Self-assembly Mechanisms of Peptide Amphiphiles

Peptide amphiphiles behave in some respects like conventional amphiphilic molecules (surfactants and lipids) 26 in that they are observed to undergo aggregation (often into fibrils) above a critical aggregation concentration (CAC). The CAC may be detected via fluorescence methods. Pyrene is a fluorescent probe molecule that is used to locate the CAC for conventional amphiphiles, as its fluorescence is sensitive to the local hydrophobic environment 27,28. It has also successfully been used to determine the CAC for several PA systems 12,21,29–35. In contrast to this method, the fluorescence of thioflavin T is dependent on the formation of amyloid-like structures (*β*-sheet fibrils) 36,37 and has been used for amyloid fibril-forming peptides. We have recently shown that it can be used to determine the CAC of lipopeptide PAs, the value obtained being similar to that obtained from pyrene fluorescence techniques 30,33,34. Other fluorescent dyes such as Nile red have been used to determine the CAC of amyloid-forming peptides 38,39 but have not yet been widely employed in studies of PA systems. The fluorescent probe 1,6-diphenyl-1,3,5-hexatriene has been used to locate the CAC of lipopeptides 24.

In principle, other physicochemical methods sensitive to the state of molecular aggregation, such as light scattering 40,41 and proton NMR solubility measurements 42,43, may also be used to determine the CAC. The CAC will, for conventional surfactants, depend on the hydrophile–lipophile balance, although in addition, for PAs, it will be influenced by hydrogen bonding as well as electrostatic interactions. A detailed model for this is still lacking, and this presents an interesting future challenge.

The kinetics of fibril formation by PAs may be followed using techniques well established for amyloid peptides such as the aforementioned thioflavin T fluorescence 44,45. Among many examples, the kinetics of fibrillisation of *β*-hairpin peptides induced by changes in pH or ionic strength has been tuned through sequence control within the peptides, leading to control over hydrogelation kinetics 46–48. Hydrogelation results from the formation of a network of extended fibrils. Several recent studies have examined the mechanisms and kinetics of self-assembly of PAs into nanotubes. For example, the peptide *β*A*β*AKLVFF has been shown, through atomic force microscopy, to initially form protofilaments, which over a timescale of 10 h develop into short fibrils 49. After 24 h, extended helically twisted ribbons are observed. Finally, within 1 month, the helical ribbons close into nanotubes. The kinetics of the last process seem very slow, although not examined in detail. The remarkable reversible unwinding of helical ribbons into twisted tapes has been observed [via *ex situ* transmission electron microscopy (TEM) and *in situ* small-angle X-ray scattering (SAXS)] for the lipopeptide C_16_-KKFFVLK 50,51. The unwinding transition occurs very rapidly on heating (about a minute or less, depending on how fast the heating can be performed), but the reformation of helical ribbons is slower, taking many hours 50. The reversibility suggests that the two states are in thermal equilibrium, although the hysteresis on cooling points to the existence of an energy barrier for the ‘refolding’ transition. A lipopeptide comprising di-C_12_-Lys likewise undergoes a series of morphological transitions from fibrils to twisted tapes to helical ribbons then nanotubes upon ageing a solution at 25 °C 52. The process seems somewhat slower than for the C_16_-KKFFVLK system, taking up to 4 months for nanotubes to form. Stupp's group also observed intermediate states in the transition between twisted tapes and helical ribbons for a designed lipopeptide upon dissolution in water 53. The kinetics of ribbon formation was slow, with the process taking several weeks. PA self-assembly may be switched using external fields, e.g. using photo-cross-linkable PAs 23,54–58. This can lead to extremely rapid self-assembly, although in this case irreversible. Many other triggers (reversible and irreversible) for self-assembly including changes in pH, temperature and addition of enzymes are available. This topic has been reviewed in detail elsewhere 54.

**Figure 2:**
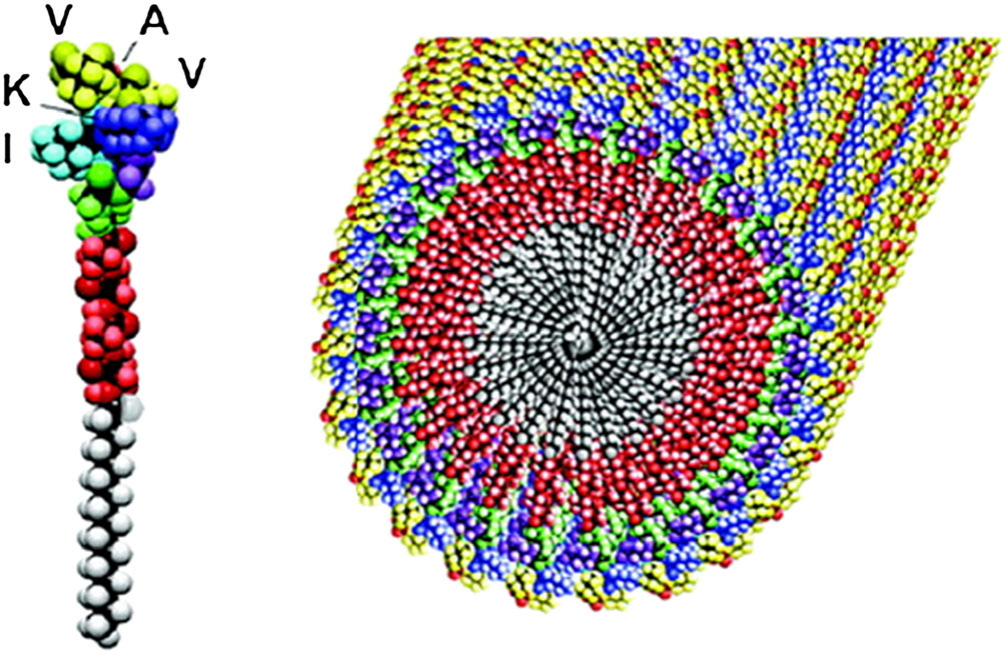
Cylindrical nanofibre formed as a result of the aggregation of individual PA molecules (with an IKVAV pentapeptide head group) above a critical aggregation concentration 8.

Lipopeptides are a class of PA that occur in nature and have defined biological functions. They include tyrosine kinase and guanine nucleotide peptides, which initiate signal transduction pathways 9,59. Other examples include lipopeptides produced by bacteria as part of their host defence mechanisms with antimicrobial and antifungal properties, among others 60. Electron microscopy has revealed that many lipopeptide molecules tend to aggregate into cylindrical nanofibres (Figure 2). In solution, PA aggregation is determined by the intermolecular hydrogen bonds between the peptide strands, which leads to the formation of *β*-sheets 3,6,8. In a system where there are only hydrophobic interactions, PA molecules would self-assemble into micelles 8. In contrast, a system with purely intermolecular hydrogen bonds would contain *β*-sheets. If a system has high hydrogen bonding energy, this will favour cylindrical nanofibres over spherical micelles. The cylindrical fibres comprise peptides lying perpendicular to the long axis of the fibre 3,8,61. The peptide epitope is presented with very high densities, which makes PAs promising biomimetic materials 4,62,63.

Paramonov *et al*. investigated the role of hydrogen bonding in the self-assembly of nanofibres 3. They discovered that the four amino acid residues nearest to the nanofibre core tend to produce *β*-sheets with intermolecular hydrogen bonds perpendicular to the long axis of the nanofibre. In contrast, amino acids further away from the core play a less important role in self-assembly and stability. This intermolecular hydrogen bonding is essential for the self-assembly of nanofibres and provides mechanical stability. Disruption of hydrogen bonds would lead to a change in geometry from cylindrical nanofibres to spherical micelles. Cui *et al*. discovered that the amino acids close to the nanofibre core, which cause *β*-sheet formation, can ultimately induce chirality in the hydrophobic tail regions 64. Studies have shown that peptide domains of PAs that consist of alternating hydrophilic and hydrophobic amino acid residues are susceptible to *β*-sheet formation. It is believed that the *β*-sheet hydrogen bonding perpendicular to the long fibril axis brings the amino acids closer, therefore preventing any twisting or bending in the morphology of the self-assembled structure. As a result, the *β*-sheets form anisometric cylindrical fibres, tapes and tubes 7,13,65.

**Figure 3:**
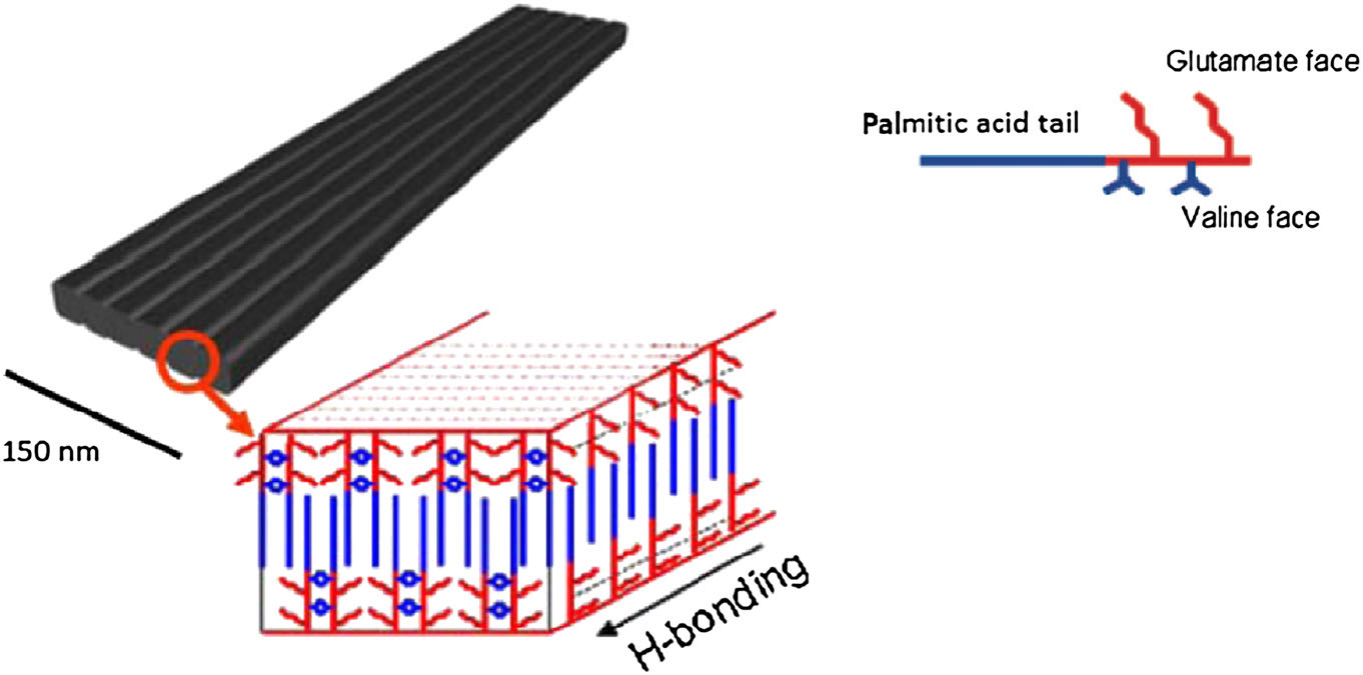
Schematic representation of the self-assembly of a PA forming a nanobelt exhibiting grooves on its surface 70.

Stupp *et al*. have reported on the self-assembly of a PA consisting of four alternating hydrophobic and negatively charged amino acids, which forms flat 1D structures termed nanobelts or nanotapes, illustrated in Figure 3 64. This type of structure is less common than nanofibres as there are two different growth rates (along the length and width directions) to consider. Another report on flat PA structures is the discovery that a pentapeptide used in an antiwrinkle cream conjugated to palmitic acid self-assembles into nanotapes (discussed in more detail later) 11.

## Cylindrical Nanofibre Self-assembly

The self-assembly mechanism of PA cylindrical nanofibres has been carefully analysed using molecular dynamic simulations as reported by Fu *et al*. 66. They report that initially, individual PA molecules begin to aggregate because of hydrophobic interactions between alkyl chains. The peptide head groups are pulled away because of electrostatic repulsion. Spherical micelles are formed because of hydrophobic interactions between the alkyl chains. A cluster of spherical micelles are formed, which begin to merge with one another because of hydrophobic interactions. This process continues, leading to a rod-like shape eventually forming a long thin fibre with the hydrophilic peptide component forming the surface and the hydrophobic alkyl chain buried within the aggregate. Lee *et al*. have investigated the self-assembly of a PA cylindrical nanofibre using coarse-grained molecular dynamics simulation 67. They observed a similar mechanism to the one reported by Fu *et al*., although spherical micelle aggregation occurred because of van der Waals interactions in contrast to hydrophobic interactions.

## Influence of the Alkyl Tail Length on Self-assembly

The addition of hydrophobic alkyl tails to PAs can enhance the thermal stability 6,9. It was discovered that a collagen-based PA that adopts a polyproline II triple helix upon self-assembly can be further stabilised by the incorporation of a hydrophobic tail 68. Further evidence for this phenomenon was revealed by using PAs of varying hydrophobic tail lengths for which the helix-to-coil transition temperature was determined. PAs with longer alkyl chains have a greater thermal stability than PAs with shorter alkyl tails 6,9. Another study showed that a peptide containing 16 amino acid residues formed a coiled-coil structure. The addition of a lipid alkyl tail induced an *α*-helical conformation with greater stability in contrast to the peptide on its own 6. Lipidation of PAs has been shown to significantly increase stability on the secondary structure of peptides, and this phenomenon can be used to produce more complex 3D PA structures.

**Figure 4:**
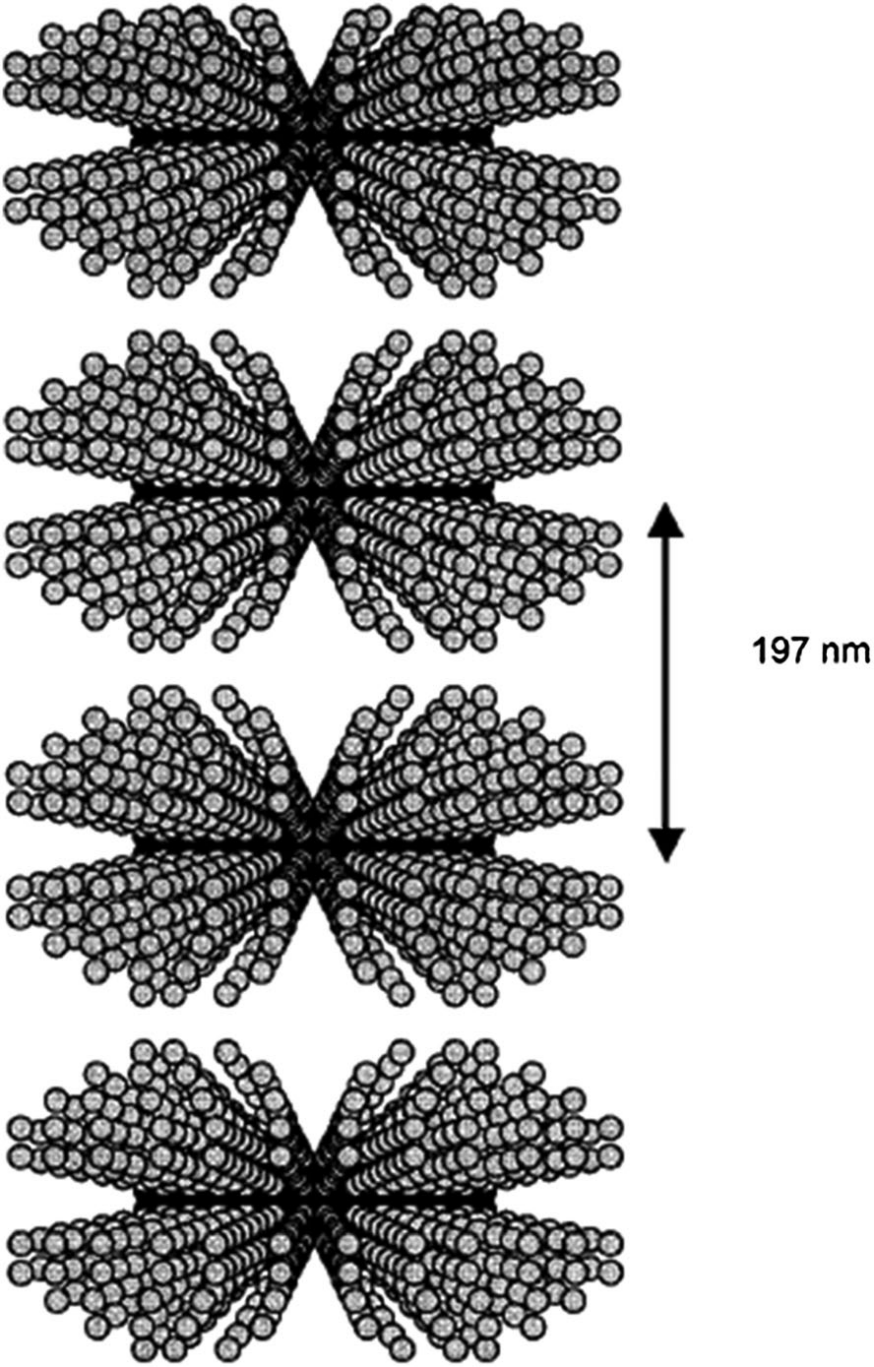
Model of stacked disc-like micelles for a PA comprising two C_14_ alkyl chains and a collagen-based peptide, consistent with fitting of small-angle neutron scattering data 77.

He *et al*. have investigated the self-assembly of a designer PA based on the amyloid-*β* peptide 69. Two different PAs were synthesised with two alkyl chains consisting of 12 carbon atoms (C_12_) attached to a core sequence from the amyloid-*β* peptide, A*β*(11–17). One variant included the attachment of two C_12_ chains attached to one side of the peptide sequence (2C_12_-Lys-A*β*(12–17)) and the other with the C_12_ chains attached to either side (C_12_-A*β*(11–17)-C_12_). Different self-assembled structures were reported for each variant. The 2C_12_-Lys-A*β*(12–17) PA self-assembled into long fibrils in contrast to the C_12_-A*β*(11–17)-C_12_ PA, which aggregated into short twisted fibrils along with lamellar structures as revealed by cryo-TEM. The Tirrell group studied the effects of the number of alkyl chains and the length of the alkyl chain attached to the (Gly-Pro-Hyp)_4_-IVH1 peptide, IVH1 being a 15-residue peptide from a type IV collagen that adopts a triple-helical structure 70. Using a combination of small-angle neutron scattering and cryo-TEM, single-alkyl-chain PAs were observed to form spherical micelles as were the double-alkyl-chain PAs. The PAs with longer tails, composed of more than 14 carbon atoms, self-assembled into disc-like micelles, which subsequently stacked to form strand-like structures (Figure 4). Hartgerink *et al*. studied the self-assembly of PAs containing CCCCGGGS[^PO^_4_]RGD(S) peptides [or controls with AAAA instead of CCCC or C-terminal sequences other than the RGD(S) cell adhesion motif] with different alkyl chain lengths 71. They observed that PAs with six carbon atom alkyl tails did not form a gel. PAs with greater alkyl chain lengths, i.e. C_10_, C_16_ and C_22_, formed gels upon acidification. At high pH the PAs have a negative charge, which prevents self-assembly owing to electrostatic repulsion. Upon acidification, the negative charge was eliminated, allowing self-assembly and hence gelation. The Cui group reported the self-assembly of a designer ABC miktoarm star peptide 72. The molecule comprises of a seven-amino-acid-long peptide (GNNQQNY) derived from the amyloid-forming region in yeast prion Sup35 linked to two hydrophobic blocks (hydrocarbon and fluorocarbon units) via a lysine junction. The miktoarm star peptide was observed to self-assemble into a mixed population of twisted and helical ribbons; however, after ageing, twisted ribbons were predominant. In contrast to the miktoarm peptide, the control molecules consisting of either a hydrocarbon or fluorocarbon unit self-assembled into nanofibres and also lacked the variety of different supramolecular structures.

## Co-assembly of Peptide Amphiphiles and the Role of Electrostatic Interactions in Self-assembly

**Figure 5:**
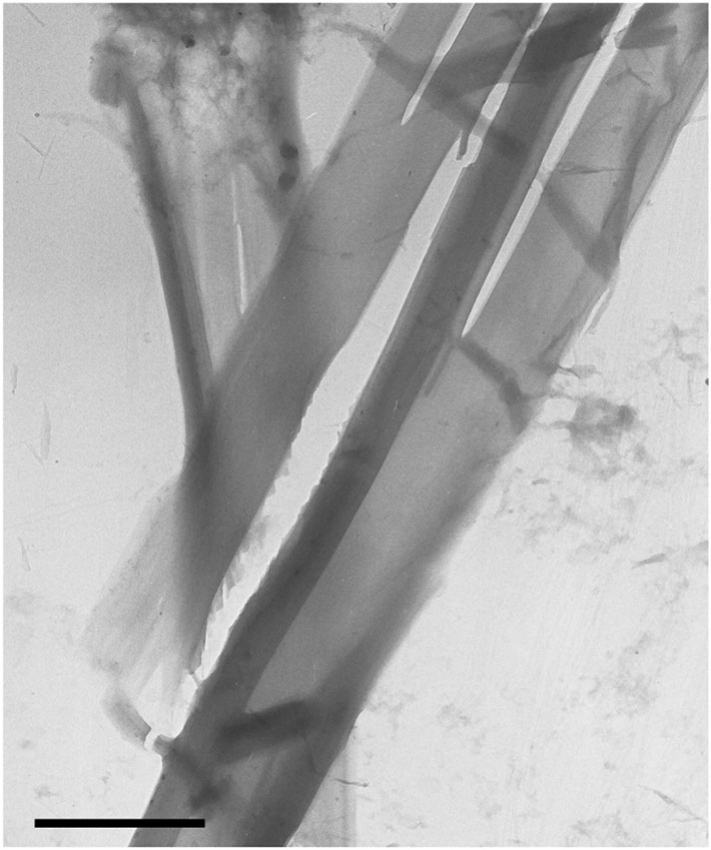
Negative stain TEM image showing tape nanostructures for a 2 : 1(wt% : wt%) C_16_-KTTKS : C_16_-ETTES mixture 83. The scale bar represents 1 µm.

Co-assembly of oppositely charged PAs can lead to distinct self-assembly behaviour as electrostatic interactions are balanced. The Stupp group has investigated the self-assembly of nanofibres produced from two PAs comprising tri-lysine or tri-glutamic acid sequences 73. They observed that the co-assembly of the PAs led to enhanced thermal stability of fibrils in contrast to the individual components as a result of electrostatic interactions between the oppositely charged lysine and glutamic acid residues. The same group also reported that a mixture of anionic and cationic PAs was able to co-assemble into nanofibres at neutral pH 74. The cationic PA component was able to self-assemble into nanofibres in basic conditions, and the anionic PA could self-assemble in an acidic solution. These observations lead to the conclusion that because the mixed system formed nanofibres, electrostatic interactions were responsible for self-assembly and not simply the hydrophobic interactions between alkyl tails. Stupp and co-workers also investigated the co-assembly of a PA C_16_-A_4_G_3_(KLAKLAK)_2_ and the same PA attached to a PEG chain 75. The PA self-assembled into cylindrical nanofibres, and the same was observed for the PA/PEG sample. The mixture was placed into a solution of hexafluoroisopropanol, which is known to disrupt hydrogen bonding to destabilise the self-assembled structure. Nanofibres with greater lengths than the individual components were formed because of co-assembly of oppositely charged molecules via electrostatic interactions. The self-assembly of designed PA C_16_-ETTES containing two anionic residues and mixtures with C_16_-KTTKS containing two cationic residues has been investigated. Co-assembly of the two PAs forming highly extended nanotapes was observed (Figure 5), along with enhanced *β*-sheet ordering as evidenced by both TEM and CD 33. The importance of electrostatic interactions in the self-assembly of PAs has been highlighted by Tsonchev *et al*. 76. Monte Carlo simulations were performed on model PAs, which were shown to self-assemble into cylindrical nanofibres as a result of both hydrophobic interactions between the lipid chains and hydrogen bonding between the peptide segments. Under specific pH conditions, large dipoles are present in the peptide head groups, which are aligned by the intermolecular hydrogen bonding. It was concluded that the cylindrical nanofibres are stabilised by electrostatic interactions between the large dipoles present on the surface.

## Tuning Self-assembly of Peptide Amphiphiles

Fine tuning or manipulating the self-assembly of PAs by altering the local environment has proven to be useful in the production of functional biomaterials. Engineering PAs to self-assemble in a particular way in response to a stimulus has been exploited in the applications of drug delivery systems, cell culture media and biosensing. The self-assembly of switchable PAs can be initiated by incorporation of photocleavable or photo-cross-linking units 23,56, changes in temperature 43, changes in pH 69,71,77,78 or the action of enzymes on PAs incorporating suitable enzyme substrates 50,79,80. These are discussed in turn in the following.

### Light-responsive Peptide Amphiphiles

**Figure 6:**
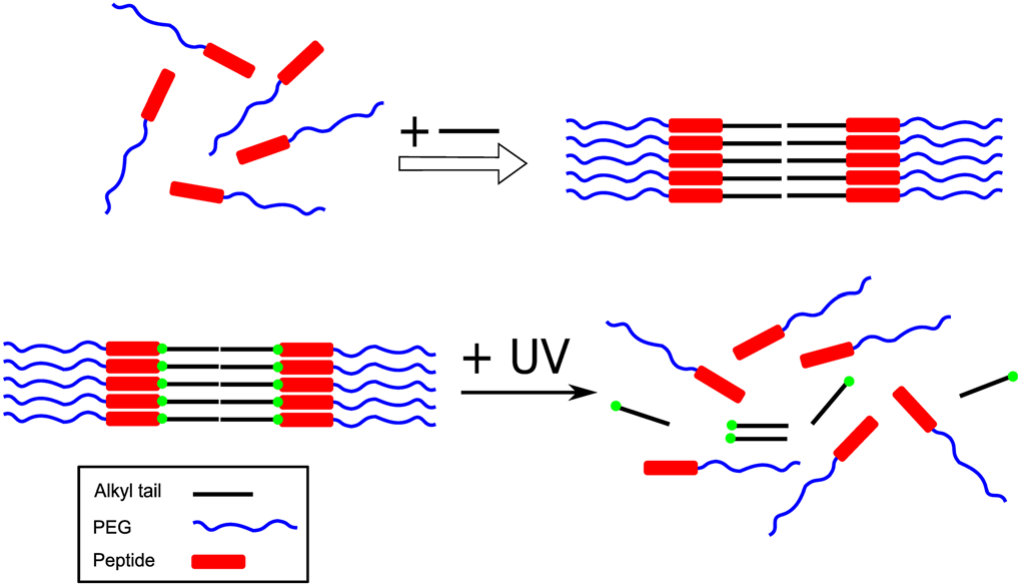
Introduction of an N-terminal alkyl chain enables the self-assembly of a PEG/peptide containing an N-terminal KTVIIE sequence along with a photocleavable linker (top); exposure to UV light then causes disassembly (bottom) when the alkyl chain is cleaved 92. Courtesy of D. Löwik.

The disassembly of fibres made from an amyloid-forming hexapeptide (Ac-Lys-Thr-Val-Ile-Ile-Glu-NH_2_), which was attached to an alkyl chain by a UV-responsive nitrobenzyl moiety, has been examined 81. Exposure of the PA to a strong UV source led to a transition from a *β*-sheet structure to a random coil as revealed by CD. The nitrobenzyl group, which acts as a linker, is cleaved in the presence of light, which separates the alkyl chain from the oligopeptide, causing disassembly of the fibres (Figure 6). The Stupp group has studied the self-assembly of a PA that forms a clear solution and that forms a hydrogel when exposed to UV light 23. The designer PA was composed of an alkyl chain and a UV-sensitive 2-nitrobenzyl group attached to a functional bioactive peptide epitope Arg-Gly-Asp-Ser. In the absence of light, the PA self-assembles into nanospheres as elucidated by TEM. When exposed to light, the PA self-assembles into a long extended network of fibres forming a hydrogel. Photo-irradiation occurs at 350 nm, which leads to the cleavage of the nitrobenzyl group causing the sol-gel transition. The same group also investigated another PA that responds to a light stimulus 82. They designed a PA consisting of a lipid chain with a 2-nitrobenzyl group attached to a peptide sequence GV_3_A_3_E_3_. They discovered that the PA self-assembles into quadruple helices, and upon light exposure, the helices dissociate into single fibres owing to photocleavage of the nitrobenzyl group.

### Temperature-responsive Peptide Amphiphiles

**Figure 7:**
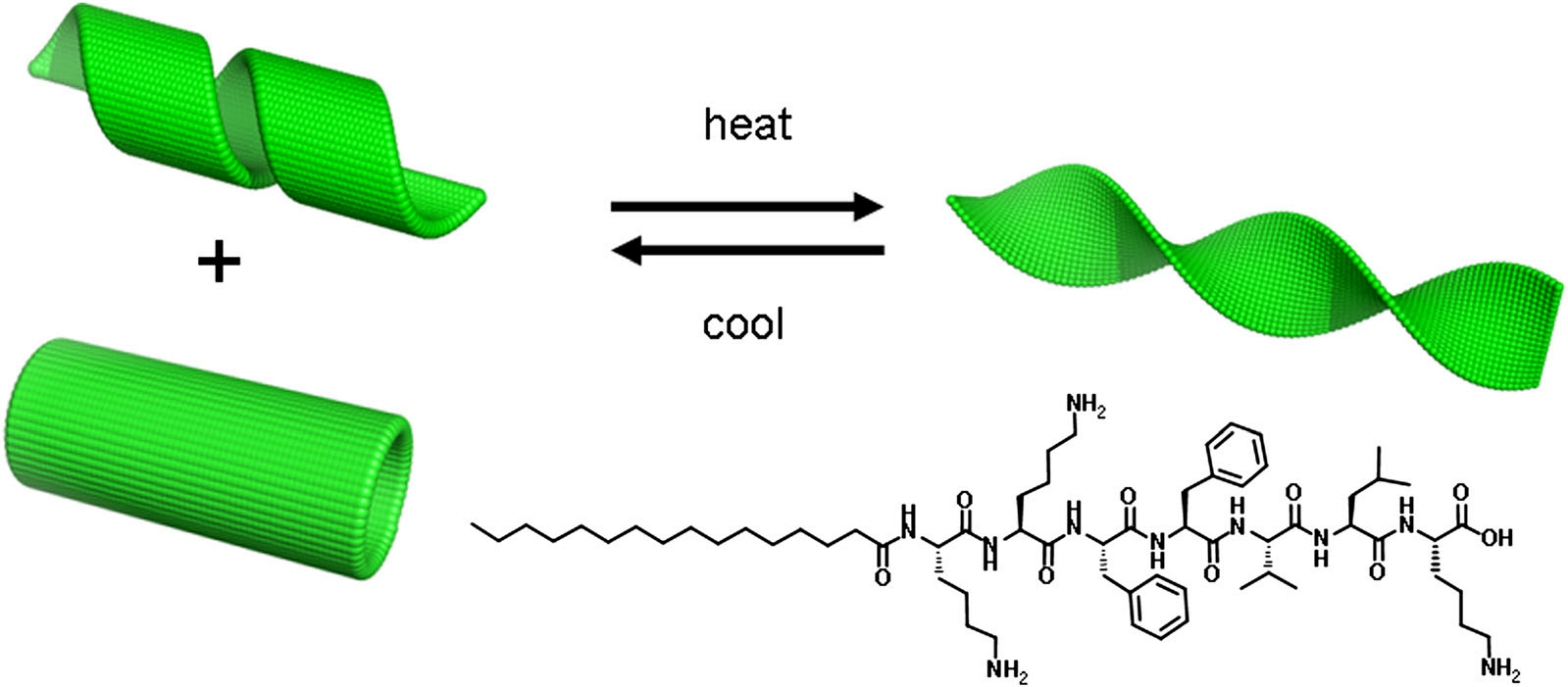
Thermoreversible transition between nanotubes/helical ribbons and twisted tapes for the PA shown 11.

Temperature effects on the self-assembly of PAs have been investigated in our laboratory 56. The self-assembly of a collagen-stimulating PA, C_18_-KTTKS, was investigated. At room temperature, the PA adopted a *β*-sheet structure as indicated by CD spectroscopy. Heating the sample above the lipid-chain melting point (55 °C) leads to a spectrum characteristic of a random coil. Recooling the solution was found not to immediately restore the *β*-sheet structure, but a few hours were required for an ordered structure to be reformed. The thermal treatment of C_16_-KTTKS has also been studied 43. It was reported that the PA self-assembles into nanotapes at 20 °C. After thermal treatment, a morphological transition into spherical micelles at 55 °C occurs, and upon recooling, the nanotape structure is retained. A PA incorporating a peptide based on the amyloid-*β* peptide KLVFF (C_16_-KKFFVLK) was observed to undergo a thermoreversible unwinding transition (Figure 7) 51. At room temperature, the PA self-assembles into helical ribbons coexisting with nanotubes as revealed by cryo-TEM. Heating at 55 °C causes a transition to into twisted tapes. Interestingly, it was discovered that the helical ribbons/nanotubes reformed upon cooling. The reversible transition was analysed by CD spectroscopy, which revealed a decrease in ellipticity at 55 °C in the vibronic transitions of the phenylalanine range (240–280 nm). During cooling, an increase in ellipticity was observed. It was reported that the thermoreversible transition is associated with changes in the aromatic interactions between the two phenylalanine residues.

### pH Effects on Peptide Amphiphile Self-assembly

**Figure 8:**
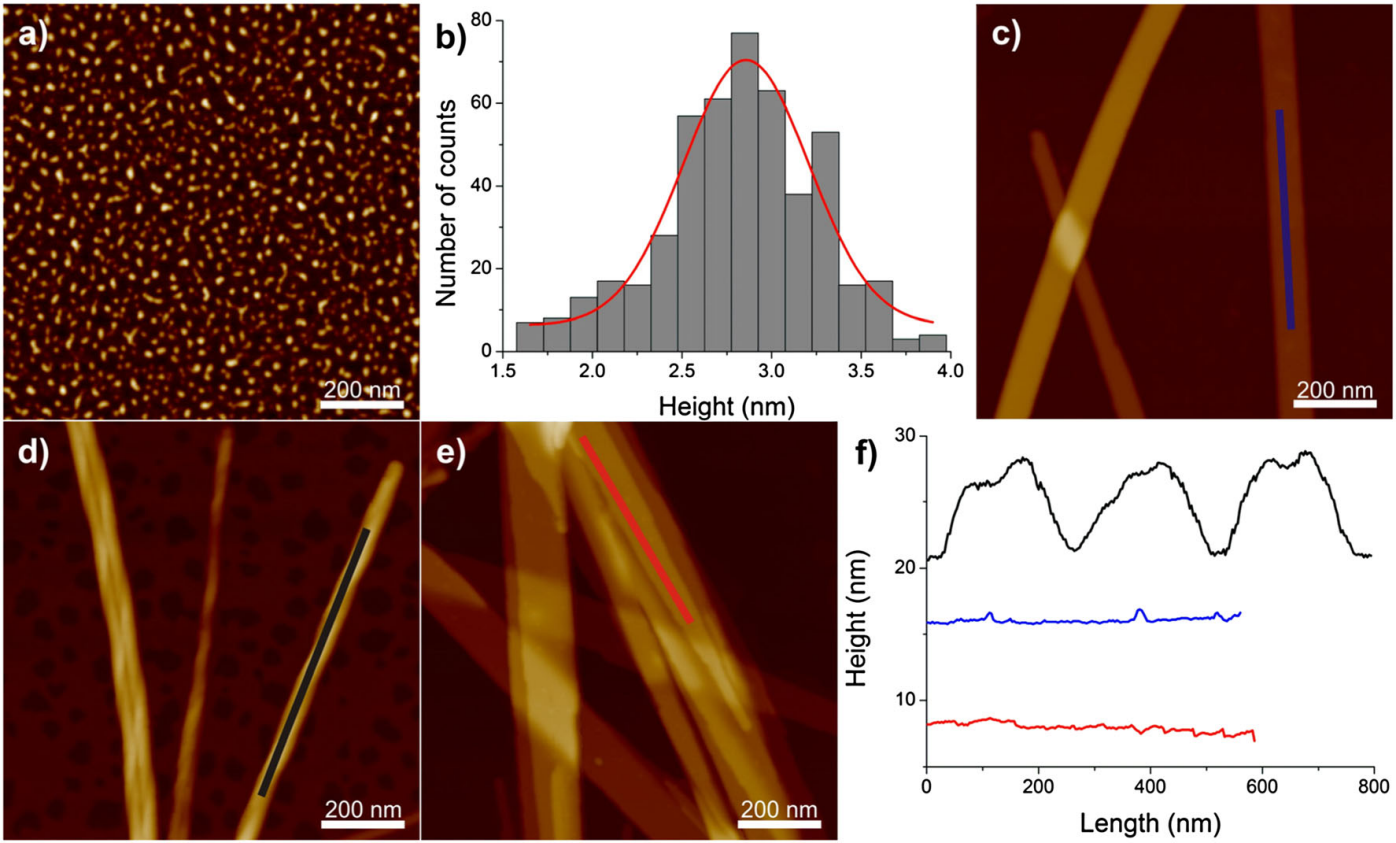
Dependence of morphology of C_16_-KTTKS on pH 10. (A) Atomic force microscopy (AFM) image of 1 wt% C_16_-KTTKS at pH 2. (B) The height distribution for spherical micelles extracted on the AFM images of 1 wt% C_16_-KTTKS at pH 2. AFM images of 1 wt% C_16_-KTTKS at (C) native pH 3, (D) pH 4 and (E) pH 7. (F) The longitudinal height profiles of the structures observed for 1 wt% C_16_-KTTKS at native pH 3 (blue curve), at pH 4 (black curve) and at pH 7 (red curve). *Z* scale for the AFM images is 6 nm for (A) and 60 nm for (C–E).

A change in solution pH can influence the self-assembly of PAs. Our group recently investigated the pH effects on the self-assembly of C_16_-KTTKS 10. The self-assembled structures were investigated at pH 2, 3, 4 and 7. A 1 wt% solution was measured to have a pH of 3, and nanotape structures were observed. Interestingly, the nanostructures change considerably with pH reductions within the same system, from flat tapes to twisted right-handed fibrils back to flat tapes and then spherical micelles (Figure 8) 10. A morphological transition by altering the pH was also reported by Deng *et al*. 83. They studied a PA that was designed based on the amyloid-*β* peptide and that self-assembles into nanofibrils at pH 3 and nanoribbons at pH 10. The Stupp group has studied the self-assembly of C_16_-RGD [C_15_H_31_C(O)-CCCCGGGS(PO_4_^2−^)RGD] based on the RGD cell adhesion motif, which aggregates into fibres at pH 4 and disassembles at higher pH 71. Guo *et al*. have reported that the PA C_12_-GAGAGAGY can form nanofibres at pH 11 and bundles of nanoribbons at pH 8 owing to hydrogen bonding between the COO^−^ and phenolic hydroxyl groups on either end of the nanoribbons 84. Hydrogels responsive to pH have been reported by Lin *et al*. 85. At high pH, the PA studied (C_16_-GSH) self-assembles into rods and ribbons. As the pH decreases to 4, a network of entangled fibres is observed, which leads to the formation of a hydrogel.

### Enzyme-responsive Peptide Amphiphiles

Several groups have demonstrated the use of enzymes as external stimuli to induce PA self-assembly to create hydrogels and drug delivery systems 79,80,86,87. Enzymes are natural biological catalysts, which carry out a variety of biochemical reactions to sustain life. Under physiological conditions, they have excellent specificity and activity. Many different enzymes exist in nature, performing a variety of functions. The main enzymes that have been utilised for PA self-assembly include the proteases (e.g. *α*-chymotrypsin) and phosphatases 88. Fine tuning of the self-assembly of PAs containing aromatic residues can be achieved by the presence of *α*-chymotrypsin as the enzyme can catalyse the hydrolysis of peptide bonds on the C-terminal side of aromatic residues such as phenylalanine 50,88. Incorporation of a functional group responsive to enzymes has been utilised to initiate the self-assembly or disassembly of PAs 89,90.

**Figure 9:**
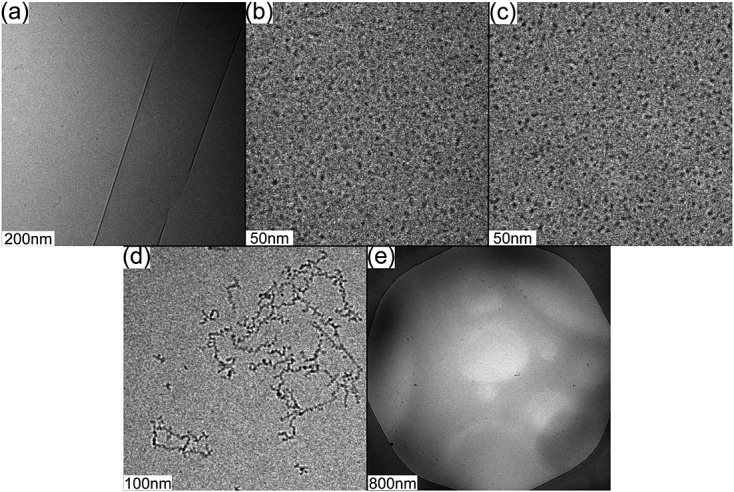
Cryo-TEM images for solutions containing 1 wt% of (A) C_16_-KKFFVLK, (B) C_16_-KKFF, (C) C_16_-KKF, (D) FVLK and (E) VLK 50.

The Stupp group has reported an enzyme-driven process to control assembly and disassembly of a PA, KRRASVAGK[C_12_]-NH_2_ 79. The PA self-assembles into high-aspect-ratio fibres. Upon treatment with the enzyme protein kinase A (which causes phosphorylation), the fibres disassembled. The reassembly of fibres occurred following treatment with alkaline phosphatase. The Kostarelos research group studied the self-assembly of a PA forming nanofibres, which was designed to internalise within neurons for applications as a drug delivery or tissue repair system 91. Because enzymes are present in circulation, the group investigated the degradation of the nanofibres susceptible to enzyme hydrolysis using peptidases. It was reported that the length of the nanofibre decreased with time (days) as disassembly occurred because of the presence of the enzyme. Our group investigated the self-assembly of a PA designed based on the amyloid-*β* peptide (C_16_-KKFFVLK) 50,51 (Fig 7). The PA self-assembles into helical ribbons coexisting with nanotubes, and when treated with *α*-chymotrypsin, spherical micelle structures are formed by the PA fragment C_16_-KKF or C_16_-KKFF (Figure 9). The serine protease cleaves the peptide bond between the two phenylalanine residues, leading to two shorter peptide fragments C_16_-KKF and FVLK. The enzyme also cleaves at an additional site between the phenylalanine and valine residues to release C_16_-KKFF and VLK. Both PAs C_16_-KKFF and C_16_-KKF were found to self-assemble into small spherical micelles.

## Surfactant-like Peptides

Peptides that consist of both hydrophobic and hydrophilic amino acids, termed surfactant-like peptides (SLPs), are able to self-assemble in a similar fashion to PAs 6,92. For example, they can aggregate into high-aspect-ratio structures while displaying bioactive peptides. SLPs are a class of amphiphilic peptide comprising a head group that is a short sequence of charged residues attached to a tail group of neutral residues 6. Pioneering work on SLPs has been conducted by the Zhang group, including A_6_D, V_6_D, V_6_D_2_ and L_6_D_2_ 93–97. Other SLPs that have been investigated include A_6_K, V_6_K, V_6_H and L_6_K_2_.

Surfactant-like peptides composed of alanine residues as the tail group tend to form stable structures because of the stronger hydrophobic interactions in contrast to other hydrophobic amino acids 92,98,99. This was supported by the work conducted by Han *et al*. 100. They studied three different SLPs, which consist of a lysine head group but with different hydrophobic tail groups: G_6_K, A_6_K and V_6_K. The peptide A_6_K was found to form stable nanotubes in contrast to G_6_K, which did not aggregate because of its weak hydrophobic character. Similar to PA self-assembly, hydrophobic interactions between the amphiphilic peptides are the main driving force for self-assembly. In addition to their surfactant-like behaviour, interstrand hydrogen bonds are formed, which can lead to high-aspect-ratio structures such as ribbons, nanotubes, nanofibres and nanorods 101,102.

### Nanotube-forming Surfactant-like Peptides

**Figure 10:**
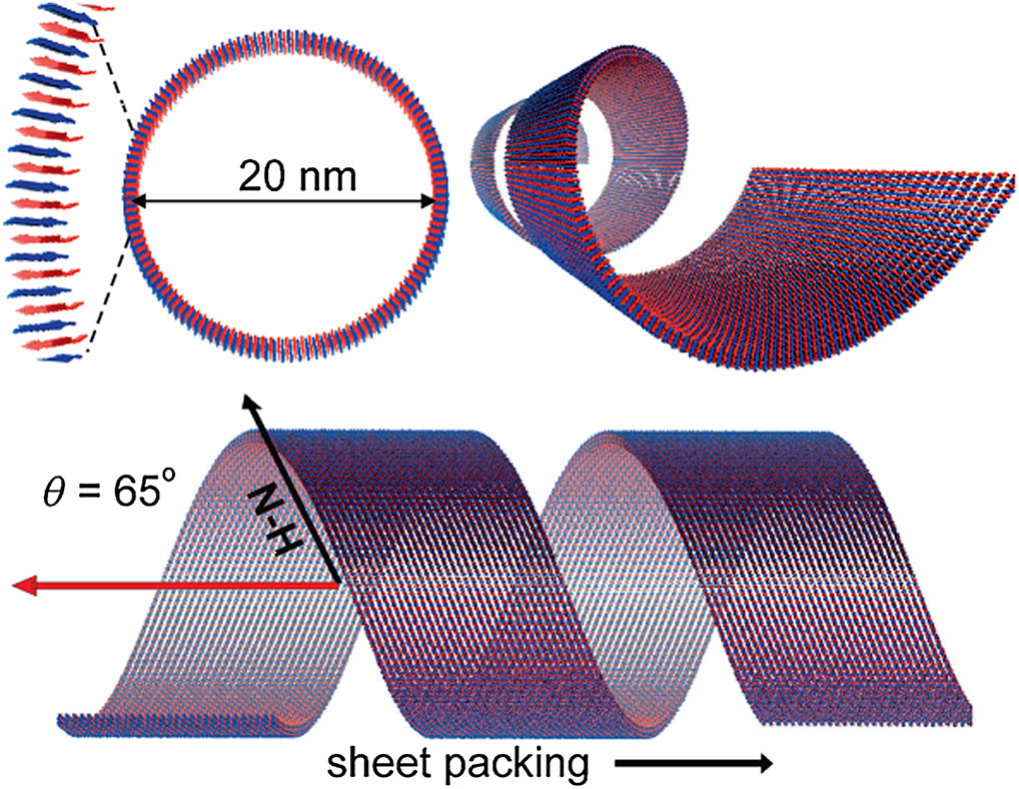
Model for the structure of A_6_K nanotubes based on information from small-angle scattering, isotope-edited FTIR and solid-state NMR experiments 104.

The first step in tube growth is the formation of bilayers of the peptide molecules, which resemble sheet-like structures. The sheets begin to roll up to form the final state tubes, which have a defined diameter and continue to grow from the edges 92,103. The self-assembly of A_6_K has recently been thoroughly examined 103,104. It was reported that the peptide self-assembles into nanotubes at high concentrations (17 wt%). Middleton *et al*. further probed the molecular structure of A_6_K 104. Fourier transform infrared (FTIR) on isotope-labelled peptides was performed, which showed an antiparallel arrangement of *β*-sheets. Solid state NMR revealed that the peptides in the nanotube walls adopt a preferred orientation. NMR line-shape analysis was carried out, which indicated that the peptide N–H bonds are tilted 65–70° relative to the long axis of the nanotube. This bond tilting is responsible for the helical arrangement of the peptides in the nanotube wall (Figure 10). The self-assembly of a cationic peptide A_6_R that consists of six consecutive hydrophobic alanine residues as a tail group with a cationic arginine head group has been examined 105. This SLP can self-assemble into ultrathin sheets at low concentrations, and at higher concentrations, the sheets wrap around to form nanotubes and helical ribbons. Our group also investigated another variant that has double the numbers of each residue, A_12_R_2_ 106. This oligopeptide self-assembles into twisted fibres, unlike its shorter variant A_6_R. The A_12_R_2_ twisted fibrils resemble amyloid-like structures but have a much smaller diameter of approximately 5 nm owing to the tight efficient packing of the simple alanine residues, which contain small methyl units. The twisting of the fibres occurs because of the antiparallel arrangement of the *β*-sheets, which minimises electrostatic repulsion of the arginine head groups.

## Amyloid Peptides

Amyloid-like peptides may incorporate sequences of hydrophobic and hydrophilic residues, leading to SLP-like behaviours. Amyloid fibrils are associated with neurodegenerative diseases due to protein misfolding 107–110. Amyloid fibrils is a term used to describe structures with the following characteristics: (i) They comprise unbranched protein fibrils, (ii) the diameter is in the range of 3–10 nm, (iii) they consist of cross-*β* structures and (iv) regions within the structure are resistant to hydrogen–deuterium exchange, proteases and denaturation as they form highly stable structures 108,110. Amyloids comprise classical cross-*β*-sheet structures (peptide strands normal to the fibril axis) that are responsible for stabilising the amyloid fibril 108,111. Many short peptide sequences including SLPs have similar structural properties to amyloid fibrils 112.

**Figure 11:**
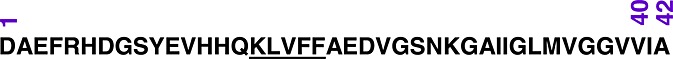
The KLVFF sequence in the A*β*40/A*β*42 primary sequence.

A short sequence in the A*β* peptide shown to be critical for fibrillisation is the amphiphilic KLVFF core motif, residues 16–20 (Figure 11) 44,113. The KLVFF sequence was found to be important in fibrillisation by Hilbich *et al*. who observed that residues 17–20 (LVFF) in the hydrophobic core region (leucine, valine and phenylalanine are nonpolar residues) aid *β*-sheet production leading to cross-*β* structures 114,115. Hilbich *et al*. determined that LVFF is indeed responsible for *β*-sheet production by substituting the hydrophobic residues with hydrophilic residues and noticed a considerable reduction in amyloid fibrillisation. It was discovered by Tjernberg *et al*. that peptides consisting of at least five residues are required to bind to a full-length A*β*40 peptide in order for fibrillisation to occur 116,117.

### Importance of Aromatic Interactions

The aromatic residues (FF) in the KLVFF fragment can form stacking interactions that are known to play a key role in amyloid fibrillisation 118. The stacking interactions have been suggested to provide an energetic contribution that enhances ordering in the self-assembly of amyloid fibrils 112,116. Diphenylalanine-based peptides have been reported to self-assemble into both vesicles and nanotubes, which highlights the importance of aromatic interactions and the role they play in molecular self-assembly 119–123.

Previous studies have shown that aromatic side chains in the tetrapeptide KFFE drive self-assembly into amyloid-like fibrils due to *π–π* stacking 124. The Phe-19 residue present in the KLVFF fragment of A*β*40 was found to be important in fibrillisation 125. Studies on other short peptide fragments containing aromatic residues, from *β*2 microglobulin and PrPC, have shown that nonbonded aromatic interactions also contribute to fibril formation 126. It is known that many amyloid fibril-forming peptides have a tendency to possess side chain aromatic residues, which are responsible for the stability of, and ordering of, fibrils 127.

Tao *et al*. have recently investigated the self-assembly of a short amyloid-*β* peptide, Ac-KLVFFAE-NH_2_, in order to study the effects of terminal capping along with varying pHs 128. It was found that terminal capping causes a change in the charge distribution of the peptide fragment and therefore has an impact on electrostatic interaction upon self-assembly. It was observed that this causes a change of morphology from nanofibrils to nanotapes via changes in electrostatic interactions. The same effect is observed when the pH is varied from acidic to basic conditions. This occurrence was explained by variation of the pH of the solution, which causes a change in the molecular packing within nanotapes in comparison with nanofibres.

### Experimental work on KLVFF-based Peptides

**Figure 12:**
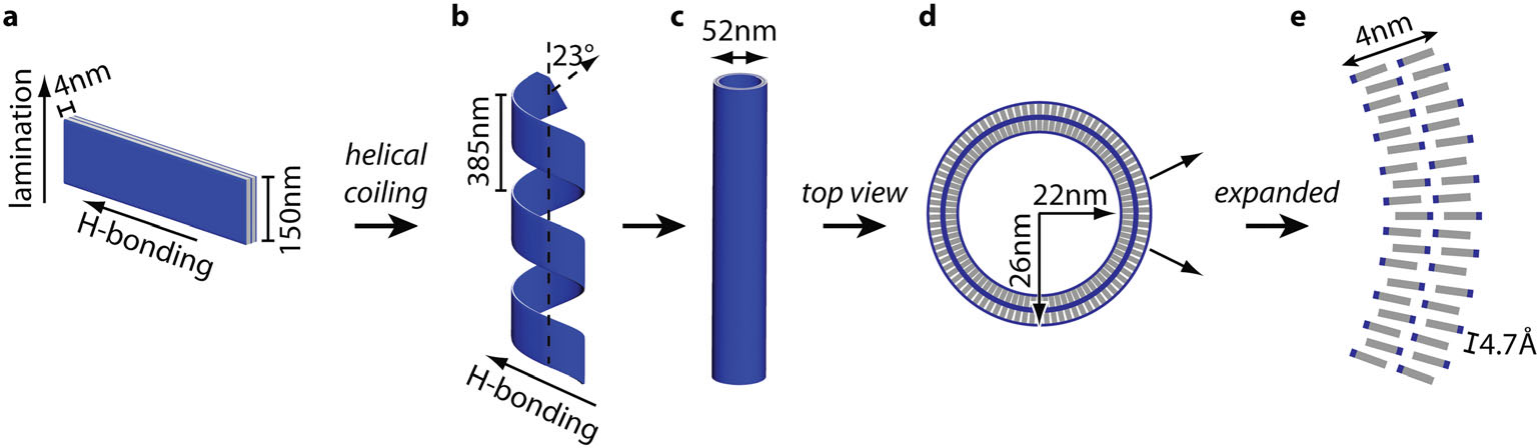
Model for the assembly of *β*-sheets of KLVFFAE into nanotubes 143. (A) A flat rectangular bilayer, (B) coiled tubular fibril with helical patch of 214 nm, (C) top view of nanotube and (D,E) details of molecular packing in nanotube wall. Figure courtesy of D. Lynn, based on Ref. 143.

Lynn's group has investigated the self-assembly of the peptide CH_3_CO-KLVFFAE-NH_2_, a sequence from the amyloid-*β* peptide, A*β*(16–22). This peptide self-assembles into nanotubes in a pH 2 acetonitrile/water solution 129,130. A model for the lamination of peptides and the curvature of peptide bilayers to form nanotubes was proposed (Figure 12), using dimensions obtained from small-angle neutron scattering experiments. The peptide forms fibrils at pH 6 131. Differences in the packing of the peptides in the antiparallel *β*-sheets (changes in strand registry) were elucidated using solid-state NMR using [1-^13^C]L17-labelled and [^15^N]A21-labelled peptides, along with MD simulations and FTIR and X-ray diffraction data 131. Isotope-edited FTIR using V18 congeners provided additional information on strand registry 132. The formation of a salt bridge between K16 and E22 plays an essential role in the formation of fibrils at pH 6. This was confirmed because KLVFFAL formed nanotubes at both pH 2 and pH 6 131.

Fluorescence labelling of this peptide at the K residue with a rhodamine dye enabled the nucleation and growth process, upon dissolution of the peptide of amyloid assemblies, to be imaged 133. Nucleation from a higher temperature state of molten globules has also been examined, with intermediate ‘necklace’ structures being observed 134. Bundling of KLVFFAE nanotubes into lamellar-like arrays can be induced by ‘salting out’ using sulfate anions 135. The congener peptide KLVFFAL also forms nanotubes, and solid-state NMR was used to probe the bilayer ordering within the nanotube walls 136. The bilayer leaflets are coated with the TFA counterions bound to lysine residues. The binding of the amyloidogenic dye Congo red into the grooves of this peptide occurs in an aligned manner along the nanotube wall (offset from the nanotube axis due to the helical packing arrangement) 137.

Krysmann *et al*. investigated the self-assembly and hydrogelation of the A*β*(16–20) fragment, KLVFF 113. This study showed, via UV spectroscopy and CD, that self-assembly in solution is driven by aromatic interactions/*π–π* stacking in which the two phenylalanine residues are responsible for aggregation. At higher concentrations, *β*-sheet formation can be observed by FTIR spectroscopy. Hydrogelation was also observed at high concentrations in phosphate-buffered saline owing to screening of the electrostatic charge on the peptide fragment.

The addition of two phenylalanine residues (FF) to the N terminus of the KLVFF fragment was found to lead to the formation of fibrils in methanol due to aromatic interactions; however, no fibrillisation was observed in water because of its high levels of hydrophobicity 138. Another variant of the KLVFF fragment, AAKLVFF, was investigated. This fragment was shown to be soluble in both water and methanol 139,140. In water, AAKLVFF forms twisted fibrils, and in methanol, AAKLVFF was observed to self-assemble into nanotubes as the *β*-sheets have the ability to twist in a helical conformation 139,140.

The incorporation of *β*-alanine residues in the N terminus of KLVFF has been studied by Castelletto *et al*. 141. The *β*A*β*AKLVFF fragment, when dissolved in water, was shown to form helical ribbons as discovered by cryo-TEM. X-ray diffraction, CD and FTIR confirm the presence of *β*-sheets, which is associated with the helical ribbon structure.

The solution self-assembly of the peptide/PEG conjugate FFKLVFF-PEG has been studied 142. The additional phenylalanine residues cause an increase in hydrophobicity, making the peptide itself insoluble in water but soluble in methanol although the PEG conjugate is soluble in water. Both optical and atomic force microscopy reveal a fibrillar structure for FFKLVFF-PEG. The presence of *β*-sheets was confirmed by FTIR, with a peak in the amide I band. X-ray diffraction was also performed, which revealed multiple reflections corresponding to the existence of *β*-sheets. A double equatorial reflection was produced, which indicated stacking of *β*-sheets perpendicular to the fibril axis.

The self-assembly of a conjugate of PEG attached to *β*A*β*AKLVFF has been examined, with potential in drug delivery 6,80. The conjugate *β*A*β*AKLVFF-PEG self-assembles into spherical micelles. In the presence of *α*-chymotrypsin, the spherical micelle breaks up into fragments of *β*A*β*AKLVF (which do not fibrillise) and F-PEG, as the enzyme cleaves bonds between aromatic residues. The peptide contains *β*-alanine residues, which are resistant to enzymatic cleavage.

## Applications of Peptide Amphiphiles

### Peptide Amphiphiles Used in Skincare Products

Peptide amphiphiles are used in collagen-stimulating products commercially available for skincare applications. A particular PA known as Matrixyl (trade name) is a lipopeptide C_16_-KTTKS 19,21,143–145. The pentapeptide used in C_16_-KTTKS is a sequence derived from type I collagen 146; however, its mechanism of action is not fully known. This lipopeptide is widely used in cosmetic applications such as antiwrinkle creams and can potentially be used in applications in regenerative medicine and wound healing 6,145.

The original study conducted by Katayama *et al*. found that the peptide sequence KTTKS could increase skin ECM production 146. The ECM is the outer region of a cell, which provides structural support for cells as well as other important functions. It is known that an immune mediator molecule called transforming growth factor *β*1 (TGF-*β*1) can cause ECM production. When TGF-*β*1 is present with KTTKS, there is a significant increase in fibroblast activity, which in turn increases skin ECM formation, therefore exhibiting a synergistic effect. Recently, it was reported that C_16_-KTTKS can enhance collagen production in a concentration-dependent manner close to its CAC (0.002 wt%) 21. The palmitic acid (C_16_) chain, which is attached to the N-terminal lysine through an amide bond, assists in the solubility to further enhance skin permeability.

**Figure 13:**
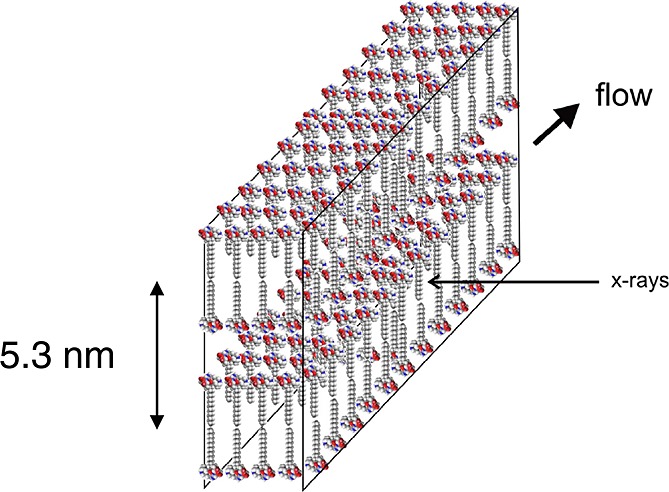
Schematic of the bilayer structure produced in C_16_-KTTKS self-assembly 11.

The self-assembly of C_16_-KTTKS has been extensively investigated by the Hamley group. C_16_-KTTKS can self-assemble into a giant tape-like superstructure with a length of tens of microns but with nanoscale width 11. Both FTIR and X-ray diffraction indicated that the nanotapes consist of a parallel *β*-sheet structure. CD confirmed the existence of *β*-sheet structures. SAXS was employed to further probe the nanostructure of C_16_-KTTKS. The SAXS data exhibited a Bragg peak corresponding to a bilayer spacing of 5.3 nm. It was proposed that C_16_-KTTKS self-assembles, forming a bilayer arrangement of the PA molecules, with the functional bioactive peptide epitope exposed on the surface of the tape as illustrated in Figure 13. As the peptide epitope is expressed on the surface of the tape, it may enhance the stimulation of collagen production.

The influence of anionic and nonionic surfactants (sodium dodecyl sulfate 147 and Pluronic P123 148, respectively) on the self-assembly of a C_16_-KTTKS was also studied, in part because the PA is mixed in formulations with surfactants. Both surfactants influenced the self-assembly of C_16_-KTTKS because morphological transitions from nanotapes to fibrils were observed.

Other PA molecules have been reported to possess collagen-stimulating properties. Both C_16_-GHK and C_16_-KT are believed to have similar effects to C_16_-KTTKS but are currently not used in the market. The self-assembly of these PAs has been examined in our lab 20. The SAXS profiles for these PAs were similar to C_16_-KTTKS in that they contained a structure factor peak relating to their bilayer spacing. In contrast to the nanotapes observed for C_16_-KTTKS, the PAs were reported to form crystal-like aggregates with lower *β*-sheet content.

### Antimicrobial Peptides

Short peptides with less than 20 amino acids comprising cationic residues can show antimicrobial activity even though their exact mechanism of action is still controversial. Peptides rich in arginine are known to have antimicrobial activity and have been widely studied 17,149–151. An example includes penetratin, a 16-amino-acid-long peptide derived from the Antennapedia protein of *Drosophila*. Another example includes the transcription activating peptide transactivator of transcription (TAT) from HIV-1, which has been reported to have antimicrobial properties 152,153. The TAT peptide is 11 amino acids long, and it is highly basic as it contains six arginine and two lysine residues. It was found that substitution of any of the basic residues with a neutral amino acid causes a reduction of antimicrobial activity, which arises from its ability to bind to cell membranes 151. Arginine contains a guanidinium group that adopts a planar Y shape in which the cationic charge is delocalised. Arginine can form bidentate hydrogen bonds with phosphates in lipid head groups as well as electrostatic interactions. As arginine interacts with cell membranes, this can lead to a negative curvature and subsequently to cell leakage, giving rise to antimicrobial properties. As a result, many have produced guanidinium-rich synthetic analogues for antimicrobial applications 151,152,154.

Berkov-Zrihen *et al*. investigated the antimicrobial activities of a series of synthetically designed paromomycin-based di-alkylated PAs 155. Paromomycin is an aminoglycoside antibiotic extracted from *Streptomyces krestomuceticus*. Both di-alkylated and mono-alkylated forms of paromomycin were found to be more effective as an antimicrobial agent in contrast to paromomycin on its own. Laverty *et al*. studied the antimicrobial activities of PAs containing the tetrapeptide OOWW (O = ornithine and W = tryptophan) 156. They investigated the influence of the hydrophobic tail on antimicrobial activity against a host of clinical bacteria using varying chain lengths of C_6_, C_8_, C_10_, C_12_, C_14_ and C_16_. The optimal antimicrobial activity was observed for C_12_-OOWW, which was reported to disrupt bacterial cell membranes. Makovitzki *et al*. studied the antimicrobial activity of very short PAs. They analysed the antimicrobial activity of C_16_-K*x*K where *x* = A, G, L or K 157. The PA with the most activity was C_16_-KKK, which self-assembles into small oligomers in contrast to C_16_-KLK and C_16_-KGK. This study highlighted the importance of the cationic-rich peptides as antimicrobial agents. Shorter PAs were investigated such as C_16_-KK and C_16_-K; however, both proved to be inactive. Chen *et al*. investigated the antimicrobial effects of three peptides A_3_K, A_6_K and A_9_K 158. They sought to determine whether the length of the hydrophobic and cationic residues had any effect on antimicrobial activity. They observed that A_9_K, which contains the most hydrophobic alanine residues out of the three peptides, had the highest antimicrobial activity. Recently, we conducted a study on the interaction of 1,2-dipalmitoylphosphatidylcholine (DPPC) with a short cationic peptide composed of six consecutive alanine residues with an arginine head group 159. The cationic peptide has antimicrobial properties and self-assembles into sheets at low concentrations and forms nanotubes at high concentrations. The peptide A_6_R forms *β*-sheets, which are disrupted in the presence of DPPC vesicles, and the peptide interacts with the DPPC bilayer in the vesicle walls without breaking the vesicles.

### Regenerative Medicine and Drug Delivery Systems

Reports on the use of PA nanofibres that can be applied *in vivo* originate from work conducted primarily by Stupp and co-workers 4,5,13,14. PAs can also act as therapeutic agents to treat diseases by transporting hydrophobic drugs to a specific site as they can be metabolised 4. The incorporated hydrophobic tail can travel across cell membranes and increase bioavailability, while the peptide epitope can be used to target a specific cell via a ligand–receptor complex 1. Uchegbu and collaborators investigated the self-assembly of an amphiphilic variant of dalargin and its use in drug delivery 160. The PA was reported to self-assemble into nanofibres, which had high circulation times and was able to cross the blood–brain barrier for transport to the brain. The PA nanofibres are coated with the peptide epitope in, contrast to typical PAs, in which the peptide head groups extend radially outwards. The group of Cui has reported the design of a drug amphiphile that can form stable nanostructures for self-drug delivery 161. The drug amphiphile consisted of the popular anticancer hydrophobic drug camptothecin conjugated to a peptide derived from the tau protein. The drug amphiphile was able to form fibril structures because of the interplay of hydrophobic interactions as well as intermolecular hydrogen bonding between the peptide head groups. Zhang *et al*. investigated the use of a cell-penetrating peptide, TAT, as a vehicle for drug delivery of the anticancer drug paclitaxel 162. The three synthesised TAT peptides conjugated to differing numbers of octanoic acid (hydrophobic) units. The TAT peptide conjugates were reported to consist of *β*-sheet structures, which lead to the formation of high-aspect-ratio fibres. The TAT PA containing the most octanoic acid units was able to efficiently encapsulate paclitaxel owing to the high hydrophobicity. The paclitaxel-containing nanofibres were able to transport the drug to cancer cells, making this an efficient system for drug delivery.

### Material Templating of Inorganic Structures

As discussed earlier, peptide-based amphiphiles can self-assemble into a range of structures on the nanometre scale. The ability to fine tune the noncovalent interactions provides flexibility in PA morphologies, which makes them ideal and attractive as organic templates to construct novel silica nanostructures. Biomineralisation is a natural biological process that occurs in order to harden tissue and as a result produces sophisticated and attractive inorganic structures 163,164. Many have produced inorganic silica nanostructures by mimicking the biomineralisation process by utilising the sol-gel condensation of silica. One method employs tetraethyl orthosilicate (TEOS), a silica precursor, which polymerises on the outer surface of organic templates 165,166. Inorganic silica nanostructures have been used to provide versatile structural and functional materials in a wide range of areas such as electronics, catalysis and sensors as well as biomedical applications including drug delivery 167,168.

Numerous authors have reported the synthesis of silica nanostructures by exploiting the natural self-assembly process of PAs. The Hartgerink group investigated the templating effect of PAs using the sol-gel condensation of TEOS to synthesise hollow silica nanotubes 22. Five designer PAs were prepared to study the catalytic activities of amino acids on silica polymerisation. All five PAs self-assembled into *β*-sheet-forming nanofibres. The PAs that contained lysine or histidine residues were reported to produce well-defined silica nanotubes owing to the catalytic effects on their side chains. Silica nanostructures were fabricated using an amyloid-like peptide (Ac-KFFAAK-Am) as a template by the Guler group 169. The peptide self-assembled into nanofibres, which were then used as a template to create silica nanofibres. Silica templating by SLPs comprising A_6_K and V_6_K was studied by Wang *et al*. 170. A_6_K self-assembled into nanofibres, whereas V_6_K formed sheets with a lamellar stacking arrangement. Silica nanostructures were synthesised by the addition of silicic acid to solutions of peptide. The formation of flower-like, fibril and lamellar morphologies was observed. The silica templating of a short SLP, I_3_K, using TEOS has been investigated by Zhang and co-workers 171. The amphiphilic peptide was reported to self-assemble into nanotubes and therefore utilise the sol-gel condensation reaction of TEOS was used to produce silica nanotubes.

## Summary and Conclusions

In summary, the self-assembly of amphiphilic peptides leads to a variety of nanostructures among which nanofibrils are the most commonly observed. These structures are stabilised by both hydrogen bonding and the hydrophobic effect. Nanotape or nanotube structures are observed when the hydrogen bonding interactions predominate whereas micelles occur when hydrophobic interactions predominate. Whether nanotapes or nanotubes form depends on a balance of intermolecular forces, in particular the tendency of the constituent *β*-sheets to twist. We discussed an example of a lipopeptide system in which self-assembly in one or the other structure can be reversibly induced by changing the temperature.

In lipopeptides, the alkyl chain(s) contribute strongly to the hydrophobic interactions, providing a means to tune self-assembly through chain length. The balance between hydrophobic and hydrophilic sequence chain lengths will also have a crucial role in controlling the self-assembly of designed sequenced SLPs and bio-inspired/biomimetic amphiphilic amyloid peptides. Physicochemical parameters such as pH, temperature and concentration all influence self-assembly by modulating the interaction between the peptide moieties because these parameters influence electrostatic interactions and others such as *π–π* stacking interactions between aromatic residues, which plays an important role in the self-assembly of the amyloid-*β* peptide, as highlighted here, among others. Lipopeptides incorporate distinct bio-based motifs (i.e. lipid chains and peptides), whereas SLPs and amyloid peptides are purely amino acid based. This means these latter classes of self-assembling peptide are compatible with genetic engineering methods and may be expressed using the native translation system. This may be useful in the bio-based production of these materials and their ultimate scale-up.

Responsive amphiphilic peptides that respond to triggers including thermodynamic and solution equilibrium conditions, UV light or enzymes have been designed, based on distinct self-assembled structures. Several remarkable examples from each of these classes have been presented here, which provide a means to create novel bioactive functional materials.

Some lipopeptides are already in commercial use, e.g. in skincare products incorporating collagen-stimulating lipopeptides. Applications in regenerative medicine and other areas of biomedicine such as the development of antimicrobial materials are currently being explored and developed. Self-assembling peptides also offer unique possibilities in (bio)materials science, e.g. in biomineralisation and the development of biocompatible electronic and sensing materials among others. This review, while by no means exhaustive, has given a brief flavour of some of the exciting research being pursued in these directions.
